# Smart Sensing and Adaptive Reasoning for Enabling Industrial Robots with Interactive Human-Robot Capabilities in Dynamic Environments—A Case Study

**DOI:** 10.3390/s19061354

**Published:** 2019-03-18

**Authors:** Jaime Zabalza, Zixiang Fei, Cuebong Wong, Yijun Yan, Carmelo Mineo, Erfu Yang, Tony Rodden, Jorn Mehnen, Quang-Cuong Pham, Jinchang Ren

**Affiliations:** 1 Department of Electronic and Electrical Engineering, University of Strathclyde, Glasgow G1 1XW, UK; j.zabalza@strath.ac.uk (J.Z.); yijun.yan@strath.ac.uk (Y.Y.); carmelo.mineo@strath.ac.uk (C.M.); jinchang.ren@strath.ac.uk (J.R.); 2 Department of Design, Manufacture and Engineering Management, University of Strathclyde, Glasgow G1 1XJ, UK; zixiang.fei@strath.ac.uk (Z.F.); cuebong.wong@strath.ac.uk (C.W.); jorn.mehnen@strath.ac.uk (J.M.); 3 Advanced Forming Research Centre, University of Strathclyde, Renfrewshire PA4 9LJ, UK; tony.rodden@strath.ac.uk; 4 School of Mechanical and Aerospace Engineering, Nanyang Technological University, 50 Nanyang Avenue, Singapore 639798, Singapore; cuong@ntu.edu.sg

**Keywords:** adaptive reasoning, dynamic environments, human-robot interaction, path planning, robot control, smart sensing

## Abstract

Traditional industry is seeing an increasing demand for more autonomous and flexible manufacturing in unstructured settings, a shift away from the fixed, isolated workspaces where robots perform predefined actions repetitively. This work presents a case study in which a robotic manipulator, namely a KUKA KR90 R3100, is provided with smart sensing capabilities such as vision and adaptive reasoning for real-time collision avoidance and online path planning in dynamically-changing environments. A machine vision module based on low-cost cameras and color detection in the hue, saturation, value (HSV) space is developed to make the robot aware of its changing environment. Therefore, this vision allows the detection and localization of a randomly moving obstacle. Path correction to avoid collision avoidance for such obstacles with robotic manipulator is achieved by exploiting an adaptive path planning module along with a dedicated robot control module, where the three modules run simultaneously. These sensing/smart capabilities allow the smooth interactions between the robot and its dynamic environment, where the robot needs to react to dynamic changes through autonomous thinking and reasoning with the reaction times below the average human reaction time. The experimental results demonstrate that effective human-robot and robot-robot interactions can be realized through the innovative integration of emerging sensing techniques, efficient planning algorithms and systematic designs.

## 1. Introduction

The recent developments in robotics [1] and autonomous systems [2] have produced new and world-changing possibilities of integrating robotic systems into many different human activities and engineering practices. From domestic settings [3] to outer space exploration [4], and spanning across an endless number of applications in areas such as healthcare [5], non-destructive testing [6], agriculture [7], human recognition [8] and firefighting [9], there is a wide range of smart algorithms enabling the introduction of robotic systems for advanced activities previously undertaken only by humans. Indeed, smart robotic systems have the potential to perform faster and more accurately, to learn and adapt to its environment, and to make intelligent decisions [10].

However, the vast majority of existing industrial robotic systems operate with a predefined series of tasks that are planned offline. In the case of industrial robotic manipulators, the path taken by the end effector within a given workspace has proven effective for traditional mass-production processes based on repetition, but they lack intelligence or perception capability for adapting to changes in the environment [11]. Consequently, additional research and development efforts are necessary to deploy smart robotic solutions into dynamically-changing or unstructured environments, particularly for workspaces shared by independent robots and/or human workers [12].

Therefore, there is a great opportunity for industries to gain a competitive edge through the implementation of collaborative robotic systems able to interact with humans [13,14]. New and robust sensing capabilities are needed to provide robotic systems with reasoning and autonomous thinking [15,16], where these capabilities have to reliably perceive the robot workspace under real working conditions. There are numerous sensing techniques that can be adopted to perceive the robot’s surroundings, such as ultrasonic [17] and laser [18]. Nevertheless, machine vision [15] is an approachable strategy with satisfactory performance and affordable cost, based on the use of optical cameras with real-time image processing. Additionally, the introduction of new sensing capabilities requires an appropriate methodology to integrate with other robotic modules such as trajectory tracking and path planning [19] to effectively process sensing information at the decision-making level, leading to autonomous reasoning, flexibility and adaptability.

A number of existing implementations of robotic manipulators able to adapt to its environment to some degree can be found in literatures [10,20,21]. For example, in [20], the mobile robot platform Care-O-bot 3 was combined with a time-of-flight sensor, working with point clouds. On a different note, a multisensory system was proposed in [10], including industrial camera, laser and temperature sensor, where the multiple input was processed by artificial neural networks to control an industrial robot. Furthermore, in [21], they focused on the path generation in a pre-defined environment using a Lego Mindstorms EV3 manipulator arm. However, research in this area is still at its infancy, and opportunities for significant improvements exist for the development of robust manipulator systems for environments with real working conditions, beyond laboratory settings. For instance, researchers tend to select expensive sensors [20] for satisfactory accurate measurements, ignoring that this accuracy can be achieved at signal processing level. Additionally, the introduction of sensing capabilities in such systems are rough, lacking a smooth integration into the overall system [20,21]. Finally, it is also common to observe a lack of modularity in these integrated systems, where the designs are focused on particular cases and the different strategies for sensing, operation and control are linked among them. Consequently, individual functions are not easily interchangeable with other state-of-the-art technologies without significant implications on the rest of the system.

Following previous work presented in [22], an extension including more detailed analysis and evaluations is provided for a case study on the development of a robotic manipulator for interactions with a dynamically-changing environment. The robotic system is provided with sensing capabilities by means of a low-cost machine vision module, such that it is able to perform pick-and-place operations through path planning [19] with collision avoidance in real time. The overall system comprises of three independent and changeable modules: (i) machine vision, (ii) path planning and (iii) robot control, running in parallel for efficient performance. This design leads to an effectively integrated system with wide modularity.

The KUKA QUANTEC KR90 R3100 [23], an industrial robotic manipulator found in many factories, is used for the case study. Nevertheless, the proposed system is transferable to other robots and industrial applications (e.g., the approach was also tested successfully on a small KUKA KR06 R900 robot [24]). From the experiments, the proposed system is able to efficiently operate under dynamic conditions, with reaction times faster than the average human reaction time, estimated at 180ms [20]. The results demonstrate the feasibility of the proposed approach for the deployment of industrial robots into unstructured, frequently changing environments with positive implications for human-robot interactions. Hence, the main contribution of this work can be stated as the development of a highly integrated system built up from independent and easily interchangeable modules, leading to wide modularity for future extensions, implemented, tested and validated on an industrial robot, which performance has been proven effective with reaction times faster than the human reaction time.

The present manuscript is organized as follows: Section 2 gives an overview of the proposed system and its design. Section 3, Section 4 and Section 5 present the machine vision, path planning and robot control modules, respectively. Then, Section 6 describes the experimental setup and Section 7 evaluates the performance achieved by the proposed system through simulations and a physical demonstrator, with concluding remarks drawn in Section 8.

## 2. System Overview

In this work, an integrated system based on a robotic manipulator is proposed, where the robot can perform operations in real time under dynamic conditions. Online planning is made to enable a robotic end effector to perform pick-and-place tasks within a given workspace. Such an online planning consists of moving the robot to a start (pick) position, pick a given object, transport it to a given goal (place) position and release it.

Traditionally, this is a manufacturing operation carried out through predefined tasks programmed offline, as the workspace (environment of the robot) is well structured and fixed. However, the aim here is to design a system able to work in a dynamic scenario, where the workspace can change unpredictably at any time. To simulate a dynamic scenario in the experiments, a given obstacle moving within the workspace is introduced such that it can intercept the trajectory of the robot during operation. Consequently, the system is required to perceive changes in the environment accurately and re-plan the robot’s trajectory in real-time in response to potential collisions. This behavior is critical to robots that must interact with freely changing environments in which other agents (such as humans and robots) act within the robot workspace.

Advanced perception of the world in robots is made possible by giving them the required sensing capabilities. This is possible by a sensing module that is responsible for acquiring environmental information through peripheral devices and data processing. In this work, the sensing strategy adopted is based on machine vision [15], where optical cameras are used in conjunction with image processing techniques. The resulting geometric information of the world is then interpreted and applied to decision-making processes. Here, an online path planner retrieves the geometric obstacle information and re-plans a valid collision-free path to complete the required pick-and-place task. Finally, the output from this reasoning process is sent to a controller to execute the path on the physical robot. Trajectory generation that obeys kinematic constraints of the robot is performed locally within the controller through an add-on interfacing software.

**Figure 1:**
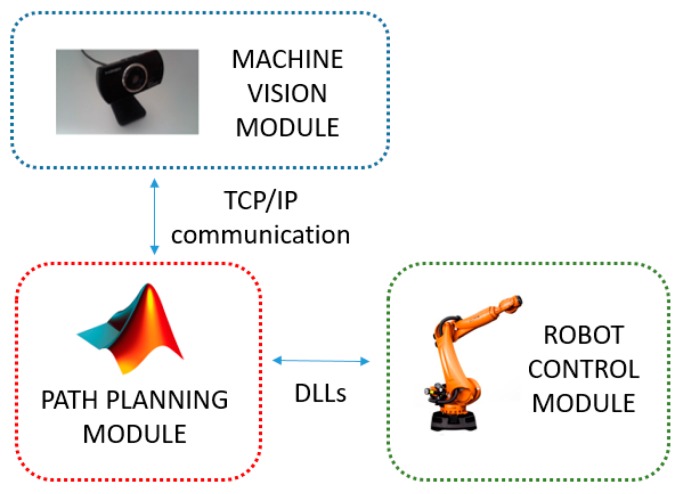
Overview of the proposed system. Three modules running simultaneously in parallel: (i) machine vision (smart sensing), (ii) path planning (reasoning, decision making), and (iii) robot control (movement coordination).

The proposed system consists of three independent modules: (i) machine vision, (ii) path planning and (iii) robot control, linked in parallel to form an efficient integrated system with wide modularity (see Figure 1). All modules work in real-time, and communications maintained across modules. The machine vision module performs obstacle detection, where dynamically-moving obstacles are tracked in the robot workspace. The decision-making process is derived from the path planning module, where the search for optimal, feasible paths for pick-and-place operations is performed based on input obtained from the machine vision to update the current geometric representation of the environment. These two modules communicate by TCP/IP sockets [25], where the machine vision software acts as a server, and the path planning module acts as a client. Finally, the resulting geometric paths are sent to the robot control module to drive the physical robot along the specified paths through Dynamic Link Libraries (DLLs). In the following sections, each of these modules will be given in detail.

## 3. Machine Vision Module

Machine vision is used to provide the robotic system with sensory attributes. This module is based on optical cameras acquiring frames in real time. The images are processed to extract relevant information about the robot environment, particularly the position of a moving obstacle in its workspace to avoid collisions.

**Figure 2:**
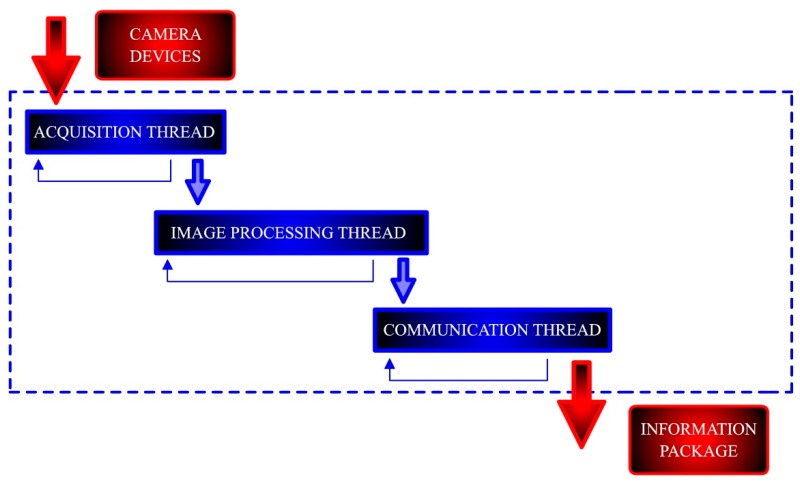
Implementation architecture for machine vision. Three different threads running in parallel for acquisition, image processing and communication. The input comes from the camera devices, while the output is stored in an information package to be sent to other modules in the system.

The machine vision is based on three independent stages: (i) frames acquisition, (ii) image processing and (iii) data communication (see Figure 2). Firstly, the acquisition stage controls the optical cameras for capturing the video stream, stating the acquisition frame rate, resolution and related parameters. Secondly, the image processing stage computes the acquired frames to perform obstacle detection. In this work, the obstacle detection is based on color with some additional filtering (by size). Finally, the last stage extracts the obstacle location obtained from image processing as information packages ready to be sent from the machine vision module when requested by the path planning module. This data transmission is based on TCP/IP sockets [25]. These three stages are implemented by three independent threads running in parallel. Consequently, the computation times for acquisition, image processing and communication do not accumulate. These are explained in the following subsections.

### 3.1. Data Acquisition

Among the different acquisition devices available for this case study, low-cost webcams were adopted to conceptually demonstrate that the enhancement of sensing capabilities can be achieved at the image processing level. In this particular case study, two cameras were placed off-board in fixed locations, instead of being mounted on the robot as in other works [26]. Off-board cameras simplify the computation of spatial coordinates in real-time and enable perception of the environment. However, this scheme can create situations in which the robotic arm invades the camera’s field of view and would hide potentially moving obstacles. For this reason, two cameras were used, and are henceforth denoted as Cam-1 and Cam-2. The main camera, Cam-1, was placed overhead, capturing a wide view of the workspace. Cam-2 was installed as a complementary camera at the side, and oriented in an orthogonal direction to Cam-1. This ensured that any moving obstacle would always be detected by at least one of the cameras, solving the robot intrusion problem for a single-camera setup. The exact location of the two low-cost cameras, as well as other considerations are discussed in the experimental setup (Section 6).

### 3.2. Image Processing

**Figure 3:**
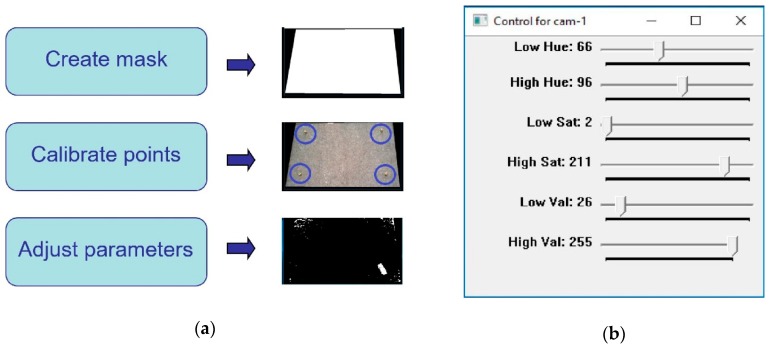
Calibration used in the machine vision module: (**a**) Main procedures for overall calibration at different stages; (**b**) Control panel window for adjustment of parameters in real time.

The frame(s) from the cameras acquired in the previous stage are then processed to perform obstacle detection in 2D (a constant height is assumed for 3D). However, initial offline calibration is required to configure the machine vision parameters. This offline calibration (see Figure 3) is carried out during the system setup, with an expected low frequency for re-calibration as lighting conditions in the workspace (and related industrial environments) are constant over time. It includes three steps. First, a given contour is defined for masking the frames, removing any information beyond the robot workspace. Then, several calibration points are measured within the workspace and taken as a reference for the computation of spatial locations. This step is necessary for the extraction of a given obstacle’s location via a projection algorithm, which translates pixel coordinates in the image to 2D real-world spatial coordinates. Finally, fine-tuning of parameters (described below) for obstacle detection is performed using a manual adjustment tool (Figure 3b), with resulting effects shown in real-time. This real-time adjustment allows an easy tuning to control a wide range of noise level hence leading to robust obstacle detection.

There is a remarkable number of potential solutions to implement obstacle (object) detection. In the up-to-date research, it is possible to find contours, descriptors and their combination [27], extensions of the correlation filter for object tracking [28], combination of color and depth (3D) images [29], probabilistic approaches to fuse multiple cameras information [30], alignment of hybrid visual features to register visible and infrared images [31], and even smart calibration procedures [32,33]. However, these techniques tend to be complex with expensive computational cost not suitable for this work.

**Figure 4:**
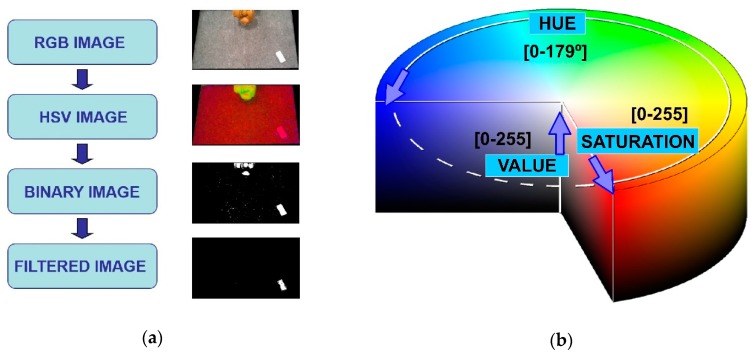
Image processing used in the machine vision module: (**a**) Main workflow for image processing; (**b**) HSV color space used for obstacle detection (scales available in OpenCV-3.1 library).

Therefore, the obstacle detection in this work is performed by color discrimination [34]. Unlike the traditional Red-Green-Blue (RGB) color space, the Hue-Saturation-Value (HSV) approach involves parametrization including not only true color (hue) but also color depth (saturation) and color darkness (value) [34], as can be seen in Figure 4. As a result, the HSV color space is much more suited for addressing real-world environments consisting of light reflections, shadows and darkened regions etc. Therefore, the real-time image processing workflow involves the following steps: (i) transformation from RGB image to HSV image, (ii) transformation from HSV image to binary image, by means of applying the selected HSV color range thresholds (one step binarization), and (iii) posterior treatment of the binary image, including size and tracking filtering, to avoid the detection of unrelated objects. This post-processing filtering is optional (HSV binarization already solves obstacle detection) and simply discards the potential presence of unrelated elements in the binary image based on their size (number of pixels in the detected region) and position in relation to threshold values empirically obtained. Figure 4 shows an example including this filtering step.

### 3.3. Communication

**Figure 5:**
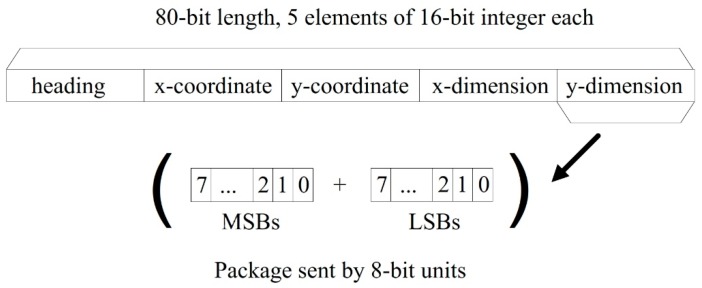
Schematic representation of the information package sent out from the machine vision module. The heading is used to avoid communication errors. Each 16-bit element is sent by two 8-bit units: Most Significant Bits (MSBs) plus Less Significant Bits (LSBs).

The communication thread is responsible for sending the latest extracted obstacle information from the machine vision module to other modules by request. As the different modules in the robotic system run in parallel simultaneously, the communication thread stores the latest information obtained from the image processing into a package and prepares it for sending as per any request by TCP/IP sockets [25]. The information package is shown in Figure 5, where it is defined by several bytes containing the position coordinates in 2D of the detected obstacle (x, y) and the approximated dimensions of the bounding box containing it. The units are in cm.

**Figure 6:**
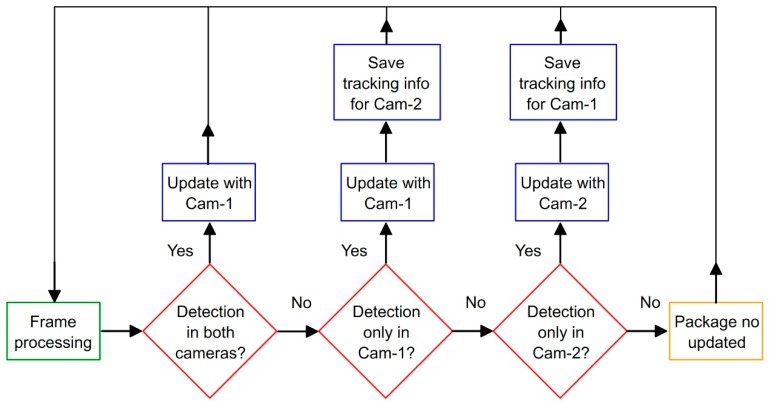
Flowchart for sensor fusion in real time [22]. Complementary camera (Cam-2) takes control when main camera (Cam-1) is not able to detect the obstacle.

Finally, an important consideration here is how to achieve sensor fusion, given that two cameras are used, with slight differences in extracted information due to sensing accuracy. The strategy shown in Figure 6 addresses sensor fusion at the output level: information from Cam-1 is always used if this camera detects the moving obstacle. When Cam-1 cannot detect an obstacle (possibly due to robot intrusion), then the information from Cam-2 is used instead. While simple, this strategy has proven effective for real-time applications.

## 4. Pick-and-Place Path Planning Module

Pick-and-place tasks are most common industrial operations in manufacturing, where a component/product is moved between predefined start (pick) and goal (place) locations within the workspace of the robot. This automation task is traditionally programmed offline, computing predefined paths which link start and goal points. However, this only works well in structured and static conditions.

By providing the robotic system with sensory attributes such as machine vision, the system is now able to interact better with its environment, adapting to dynamic and changing conditions such as a moving obstacle in the robot workspace. This interaction is enabled via a path planning algorithm that is able to interpret the sensing information and (re-) plan a globally optimal, collision-free path for pick-and-place operations in real time. Hence, when a moving obstacle invalidates an initially planned path, this path can be updated quickly and effectively.

The pick-and-place path planning approach implemented here uses the method of dynamic roadmaps, which is a sampling-based real-time variation of the Probabilistic Road Maps (PRMs) method, proven effective in motion planning within changing environments [35]. The dynamic roadmaps method is characterized by an offline pre-processing phase and an online planning and computation.

### 4.1. Pre-Processing Phase

In this phase, the algorithm creates a mapping between the states sampled in the configuration space (C-space for short) with the cells in a discretized workspace, and the sampled states are connected with their neighboring states as characterized by PRMs. This phase is carried out as follows.

**Figure 7:**
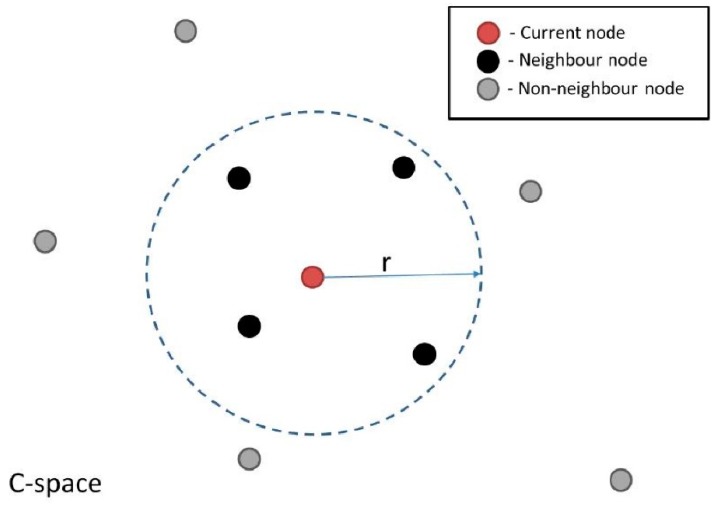
Illustration of nodes that are considered neighbors of a node being considered, with current node (red color), nodes lying within a specified radius r (black color) and all other nodes in the C-space (gray color).

Firstly, the robot C-space is sampled. This involves randomly sampling the entire C-space to obtain nodes of the roadmap, and this is performed assuming a completely obstacle-free space. Then, pairs of neighboring nodes are connected to form the edges of the roadmap. Neighboring nodes are defined as all those that lie within a predefined radius (r) from a given node (Figure 7). A single node within this roadmap represents a single robot configuration. Thus a connecting edge between two nodes corresponds to a valid motion path between two configurations.

The geometric representation of the workspace is then discretized into uniform cells, where the spatial resolution available is dependent on the cell size, with a subsequent trade-off between finer resolution and faster computation. Increasing the number of cells increases the computation time of the mapping stage (described below) exponentially.

Given the sampled C-space and discretized Cartesian space, a mapping between the two domains is performed. This mapping is obtained by iteratively checking every robot configuration associated with all the sampled nodes and along each edge of the roadmap. All workspace cells that collide with the robot at these configurations are mapped to the associated nodes and edges. Hence during online execution, the roadmap can be updated based on the cells which are occupied by obstacles, producing a graph representation of valid motions between robot configurations across the entire workspace.

### 4.2. Online Phase

During the online phase, the algorithm retrieves the perceived obstacle information from the machine vision module by TCP/IP sockets [25]. The path planning module requests and receives immediately the 80-bit package shown in Figure 5 with the latest information about the position of the moving obstacle. From this package, the algorithm knows the obstacle (x, y) coordinates within the workspace and the size of a rectangular bounding box containing it. Therefore, the obstacle is treated as a box object, providing enhanced clearance between the robot and obstacle for collision avoidance. This information is combined with the mapping computed offline to create a graph representation of the collision-free regions in the C-space.

The desired start and goal configuration (which can change at any time) is connected to the nearest node in the roadmap. Then, two steps are used to build the new path for the robot. First, the A* algorithm [36] (an extension of the Dijkstra’s algorithm for graph search) is implemented to search for the shortest route within the graph, finding a path that guarantees no collision with the moving obstacle. Then, B-splines smoothing [37] is used to smoothen the obtained path and achieve a continuous smooth motion.

**Figure 8:**
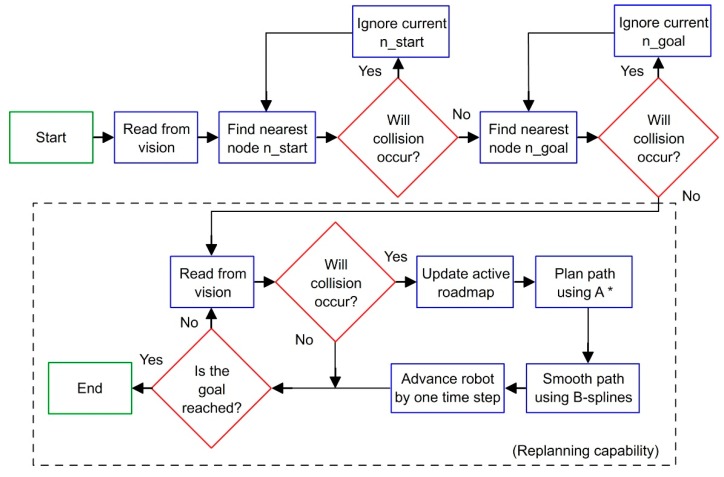
Flowchart for dynamic path planning [22]. The dashed box indicates re-planning capability.

Once the robot computes the initially planned path, the path planner continues to monitor the obstacle in the workspace. If any detected change in the environment invalidates a previously planned path, then a new updated path is computed using the steps described above. In this implementation, the algorithm is assessed for real-time performance based on its ability to plan paths faster than human reaction time, which is approximately 180ms [20]. Human reaction time is taken as reference as robots must react to changes in the environment quicker than that for a safe interaction with human workers. A high-level flowchart representing this real-time path planner is given in Figure 8.

## 5. Robot Control Module

So far, a machine vision module has been introduced for providing the robotic system with sensory capabilities, while a path planner module for decision making in pick-and-place tasks has been described. However, a third module for robot control is necessary to interface the planning results with real-time position tracking and actuator control.

Robots have been quite successful in accomplishing tasks in well-known environments like a work cell within a factory. The much harder problem of a robot acting in unstructured and dynamic environments, like those humans normally act and live in, is still an open research area [38]. In such situations, robots need to adapt their tasks after beginning an initial sequence. In this work, a novel toolbox, the Interfacing Toolbox for Robotic Arms (ITRA) [39], was used.

### 5.1. ITRA Toolbox and RSI Interface

ITRA is a cross-platform software toolbox, designed to facilitate the integration of robotic arms with sensors, actuators and software modules through the use of an external server computer. It contains fundamental functionalities for robust connectivity, real-time control and auxiliary functions to set or get key functional variables. ITRA is a C++ based DLL of functions. Due to platform availability during its development, it is currently focused around KUKA hardware, but can be extended to handle real-time interfaces on ABB [40] and Stäubli [41] robots. As such, it runs on a remote computer connected with KRC4 robots through a User Datagram Protocol (UDP/IP) socket.

All the embedded functions can be used through high-level programming language platforms (e.g., MATLAB, Simulink and LabVIEW) or implemented into low-level language (e.g., C, C# and C++) applications, providing the opportunity to speed-up flexible and robust integration of robotic systems. The ITRA is currently compatible with all KUKA KRC4 robots equipped with a KUKA software add-on known as Robot Sensor Interface (RSI) [42], which was purposely developed by KUKA to enable the communication between the robot controller and an external system (e.g., a sensor system or a server computer).

Cyclical data transmission from the robot controller to the external system (and vice-versa) takes place in parallel to the execution of the KUKA Robot Language (KRL) program. Using RSI makes it possible to influence the robot motion or the execution of the KRL program by processing external data. The robot controller communicates with the external system via the Ethernet UDP/IP protocol. The ITRA takes advantage of the fundamental functionalities of RSI and allows achieving external control of robotic arms through three different approaches.

### 5.2. Real-Time Robot Motion Control

Real-time robot motion control can be divided into two sub-problems: (i) the specification of the control points of the geometric path (path planning), and (ii) the specification of the time evolution along this geometric path (trajectory planning). Whereas the path-planning sub-problem is always dealt with by the computer hosting the ITRA, where processing of machine vision data and/or other sensor data can take place to compute the robot target position, the trajectory planning sub-problem can be managed by different actors of the system.

In the first approach, referred as KRL-based approach, the trajectory planning takes place at the KRL module level within the robot controller. The second approach has trajectory planning performed within the external computer, soon after path-planning, and is referred as Computer-based approach. The third approach relies on a real-time trajectory planning algorithm implemented into the RSI configuration. Therefore, trajectory planning is managed by the RSI context and the approach is named as RSI-based approach.

The KRL-based and the Computer-based approaches enable basic robot external control capabilities, where the robot has to wait until the current target position is reached in order to go for the next one. This means that, if a new target point C is stated while the robot is moving from a point A to a point B, the robot cannot adapt to this change until B is reached, becoming a major problem.

Unlike the KRL-based and the computer-based approaches, the RSI-based approach enables true real-time path control of KUKA robots based on KRC4 controllers. This approach, which is used for this work, permits fast online modifications to a planned trajectory, allowing robots to adapt to changes in the dynamic environment and react to unforeseen obstacles. Whereas the path-planning takes place in the server computer, trajectory planning has been implemented as an RSI configuration, employing the second-order trajectory generation algorithm presented in [43]. The approach can operate in Cartesian-space and in joint-space. While the robot is static or is travelling to a given position, the computer can send a new target position (together with the maximum preferred speed and acceleration) through a specific ITRA function. The target coordinates, received by the robot controller, are used to compute the optimal coordinates of the set point to send to the robot arm drives through a two-fold algorithm.

**Figure 9:**
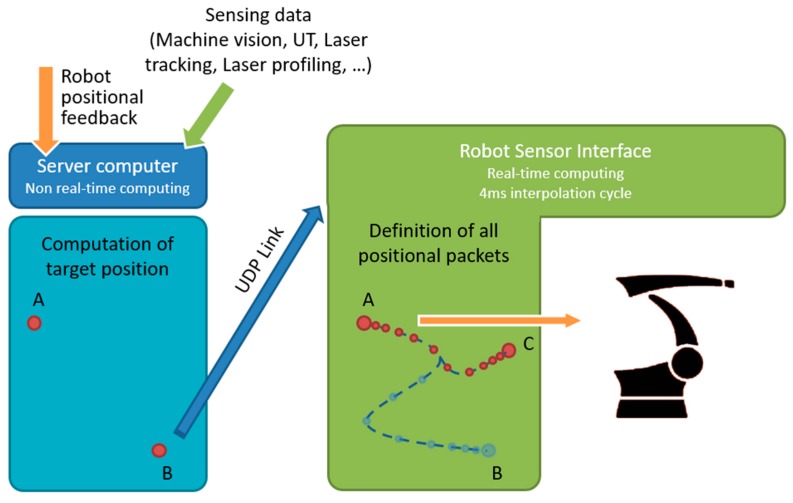
Robot Sensor Interface (RSI) for real-time computing in 4ms interpolation cycle. While moving from point A to point B, the robot can update its target position from current B to a new point C in real time.

On the one hand, the set point is generated to guarantee a smooth transition from the initial conditions (starting coordinates, velocity and acceleration) towards the final target position. On the other hand, the algorithm makes sure the evolution of the robot motion is constrained within the given maximum velocity and acceleration. Thanks to this approach, the robot motion can be quickly updated in response to the path planning module (e.g., the robot can adapt to any changes in the workspace interfering its operation). This implementation is herein referred as a robot control module and is schematically represented in Figure 9.

## 6. Experimental Setup

In this section, the experimental setup is described, defining all the components of the system including the robotic manipulator, workspace, optical cameras and moving obstacle, among others.

The experiments and related discussion in this section are specific to one case study. Nevertheless, one of the main advantages of the proposed system is its modularity and can be generally applied to different applications with no constraints on the type of robot manipulator or sensory devices used or the environment in which they are deployed in.

### 6.1. Robotic Manipulator and Pick-and-Place Elements

**Figure 10:**
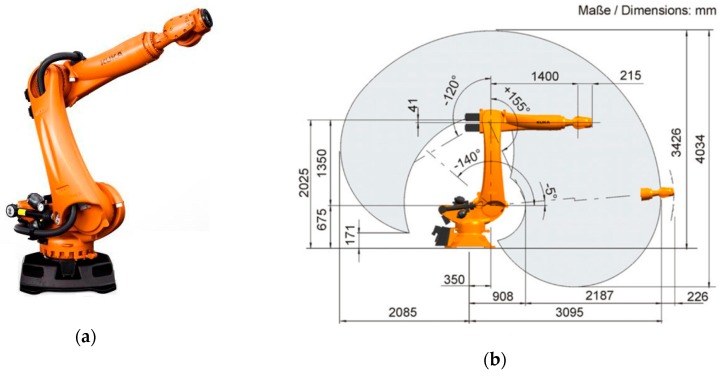
KUKA KR90 R3100 robot: (**a**) General aspect of the robotic arm manipulator; (**b**) Working envelope with dimensions comprised during operation. Images obtained from [23].

The KR90 R3100 produced by KUKA was used in this case study [23]. This robot is a 6-axis serial manipulator, with characteristics shown in Table 1 and Figure 10 [23]. This robot was chosen as it is commonly found in industrial applications. However, the proposed system can be implemented with any other serial manipulator (robots not manufactured by KUKA would require their accompanying controllers). Indeed, the KR06 R900 [24] was also employed during preliminary experiments.

**Figure 11:**
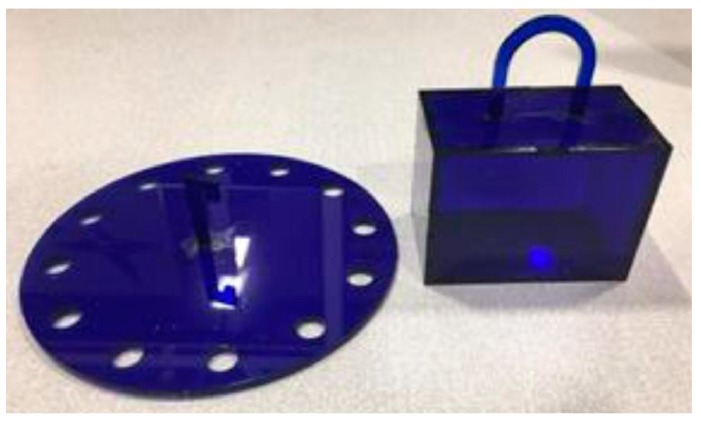
Hook and box with handle for pick-and-place tasks. The box dimensions are 10 cm × 5 cm × 5 cm and was produced using laser-cutting technology.

This robot was given the task to perform a series of pick-and-place actions guided by a collision-free motion plan. In order to perform the pick-and-place operations, a simple box and hook (Figure 11) were produced using laser-cutting technology, with the hook acting as a simple gripper. Start and goal box poses were defined for which the planner was used to plan paths to transfer the box across various targets.

### 6.2. Workspace and Moving Obstacles

**Figure 12:**
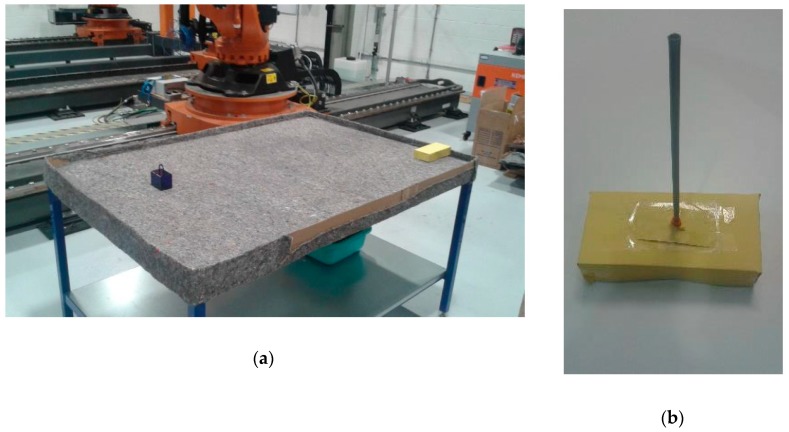
Workspace table implemented for the robot: (**a**) General overview including robot base, covered table, pick-and-place box and moving obstacle; (**b**) Moving obstacle covered in yellow color with a 30 cm ‘spike’ on its top.

A workspace for the KR90 R3100 robot can be defined according to its dimensions and disposition. In the experiments, the workspace was limited to a table with dimensions 160 cm × 110 cm × 85 cm (width-breadth-height) located next to the base of the robot (see Figure 12). The table was covered by an old, rough tablecloth, containing different textile traces and rusty/dirty patches. This, along with strong reflections and inconsistent light, simulated a noisy environment. Finally, barriers were placed along the table perimeter to constrain the moving obstacle inside the workspace.

The moving obstacle was mocked by using a remote controlled car of dimensions 20 cm × 10 cm × 5 cm covered by yellow cardboard (Figure 12b), with a speed estimated at 1 m/s. This color was chosen due to its similar tonality to the orange color of the robot, which further increases the challenge on image processing. Nevertheless, this was effectively addressed by the approach to configuring HSV parameters during calibration (Figure 3), which subsequently enables any color differences to be identified. Operator safety regulations prevented human entry into the workspace of the robot, hence the use of the remote controlled car provides reasonable dynamics within the environment. A ‘spike’ of 30 cm was placed on top to virtually extend the height of the obstacle, which contributes to greater demands on path correction. This experimental setup illustrates the robustness of the proposed system to various environmental challenges that may be present in various real-world scenarios.

### 6.3. Cameras and Location

**Figure 13:**
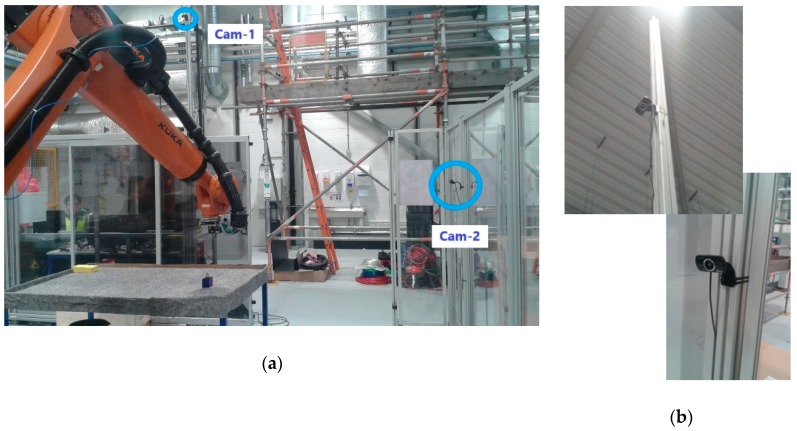
Disposition of the cameras for experiments: (**a**) Relative location of cameras with relation to the robot workspace; (**b**) Close-up view of cameras attached to cell caging.

Two HD Pro AWCAMHD15 cameras (Advent) were chosen for Cam-1 and Cam-2 (Figure 13). These are low-cost webcams able to capture images with an original resolution of 640 × 480 pixels and a frame rate of 30 fps.

The main camera, Cam-1, was placed in an overhead location (Figure 13), 3 m over the ground, capturing images of the workspace table from a landscape perspective. The complementary camera Cam-2 was placed at a much lower height of 1.5 m, orthogonal in direction to Cam-1, and therefore capturing table from a portrait perspective. The selected disposition for Cam-2 was intended for the recovery of any blind points in Cam-1 should the robot intrude Cam-1′s field of view.

### 6.4. Host Computer and Related Software

An Inspiron 15 7000 (quad-core Intel i7) laptop (DELL, Round Rock, TX, USA) with 16 GB RAM (Windows 10 operating system) was used in conjunction with a KR C4 controller to implement the proposed system. The laptop possesses 2 USB ports, one for each camera, and an Ethernet port for connection to the controller.

**Figure 14:**
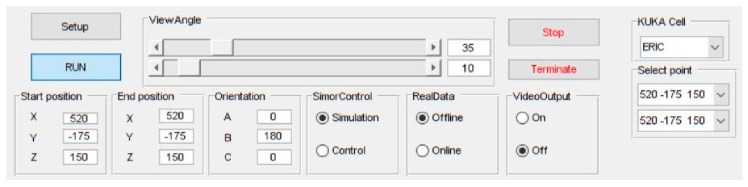
Main controls in the MATLAB GUI for the real-world physical demonstrator.

Both the path planner, implemented in MATLAB (MathWorks, Natick, MA, USA), and the machine vision, implemented in C++ and called from MATLAB through executable files, were executed on the remote PC. Actuation signals for to the controller were sent through a robot control DLL to interface with the KR C4. The integrated system was run from a friendly Graphical User Interface (GUI) also developed on MATLAB (Figure 14).

## 7. Experiments Evaluation and Discussion

In this section, the proposed system with the three described modules: (i) machine vision, (ii) pick-and-place path planning and (iii) robot control is applied to both software simulations and to a real-world physical demonstrator. Then, a performance evaluation is presented.

The proposed system was assessed in two ways. Firstly, simulations were carried out through a simulation platform developed on MATLAB and interfaced via a custom GUI. The system was then evaluated on the real-world physical demonstrator. In both cases the system performance was assessed according to its behavior in responding to the presence of dynamic obstacles and the computation time required to (re-)plan scenarios. As the obstacle detection is implemented in 2D, a constant height for the moving obstacle is assumed (30 cm ‘spike’ on its top).

### 7.1. Simulation Tool Analysis

**Figure 15:**
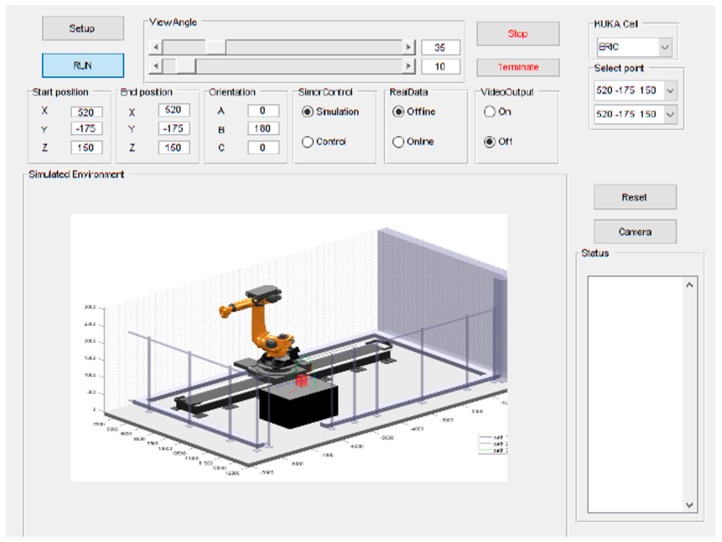
MATLAB GUI showing simulation of real-world environment for the KR90 R3100 robot performing pick-and-place tasks across table.

Prior to deployment to the real-world system, a study based on simulations was undertaken to validate the behaviors of the individual components and the overall performance of the proposed integrated system. To this end, a platform for simulations, developed in MATLAB, was integrated into a GUI for human-computer interactions (Figure 15). The simulated environment is built to exactly match the real-world setup of the physical system, where the robot performs pick-and-place path planning with motion constrained to a limited workspace around a table.

**Figure 16:**
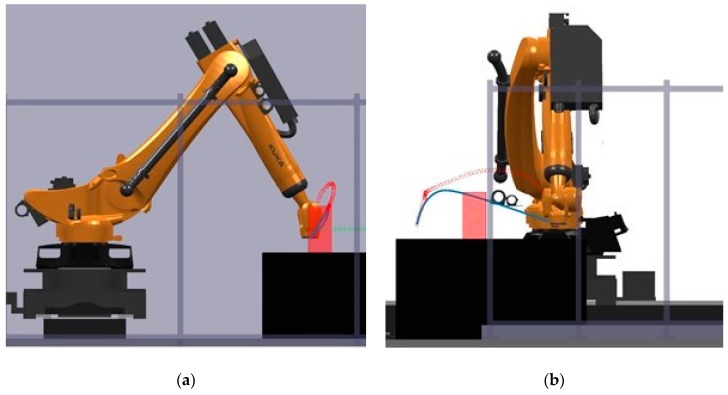
An instance of dynamic path planning simulation: (**a**) Lateral view; (**b**) Front view. The obstacle (red block) moves inwards towards the robot (path shown as a series of green crosses) as it begins executing an initially planned path (blue line). As the obstacle obstructs this plan, the planner continues to find a new feasible path, which results in the final executed trajectory shown as red circles (indicating the time evolution of the trajectory).

The simulation provided a platform to validate the behavior of the planner to respond to dynamic obstacles by considering several pick-and-place scenarios. Several unknown moving obstacle trajectories were captured using the machine vision module and used to simulate the dynamic obstacle in these simulations. The visualisation capability of the GUI enabled tracking of the changes to planned motion paths. An example is shown in Figure 16. These simulations were also used to provide an initial benchmark of the system according to its computational efficiency. Table 2 shows three different motion path problems for which the computational performance was measured and the corresponding computation times are reported in Table 3. These times are broken down according to various functions used in path planning along with the total time. Five trials for each planning problem were performed to provide statistical significance. As shown in Table 3, the computation is different for each path, even presenting some oscillations within a given path. Overall, the computation times are less than 50 ms for all trials, which meets the requirements to perform faster than the average human reaction time (180 ms) [20] by several folds.

### 7.2. Physical Demonstrator Performance

**Figure 17:**
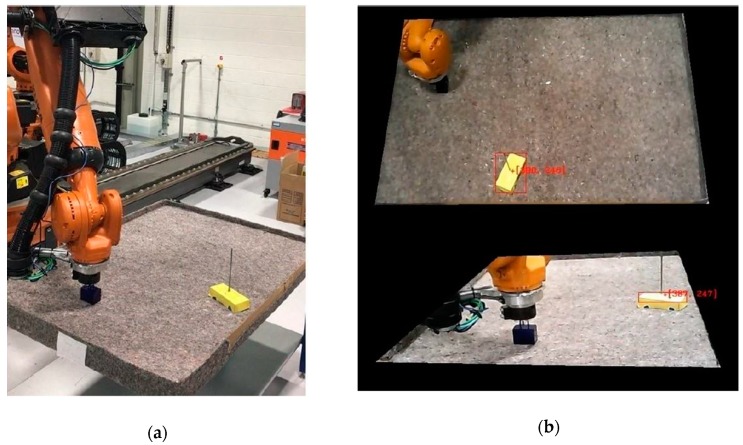
Synchronized video frames captured during operation [22]: (**a**) A real video frame taken from the safety area showing all object configurations in the environment; (**b**) Acquired frames by Cam-1 (top) and Cam-2 (bottom) from machine vision.

Trials on the physical robot showed that the integrated system provided correct behavior in response to dynamic obstacles. In all cases the robotic arm successfully performed the pick-and-place operation without collision. Two videos are provided as Supplementary files S1 and S2. Where possible, the system plans a new path to achieve the task without colliding with the obstacle. Where this is not possible (for example, when the obstacle would collide with the goal configuration of the robot), the robot waits until the obstacle is cleared away. Figure 17 provides a real video frame capturing the configurations of the objects in the environment acquired during experimentation, as well as the acquired frames from the machine vision cameras for the same time instance.

Smooth transition at switching of paths during re-planning was observed at all times. This was achieved as a result of the real-time trajectory generation implemented within the real-time control module. The successful avoidance of obstacles in all instances also validates the effectiveness of the machine vision module where, for this environmental setup, its resolution was measured to be approximately 0.3 cm per pixel. This gives an estimated error of ±3 cm in perceiving the obstacle pose, which proved sufficient for the experiments conducted. Larger image resolutions can reduce the localization error at the cost of increasing computation complexity, and this trade-off is adjustable depending on the application aims. Additionally, the speed of the moving obstacle can also affect the localization error, although no issues were found with the current setup.

**Figure 18:**
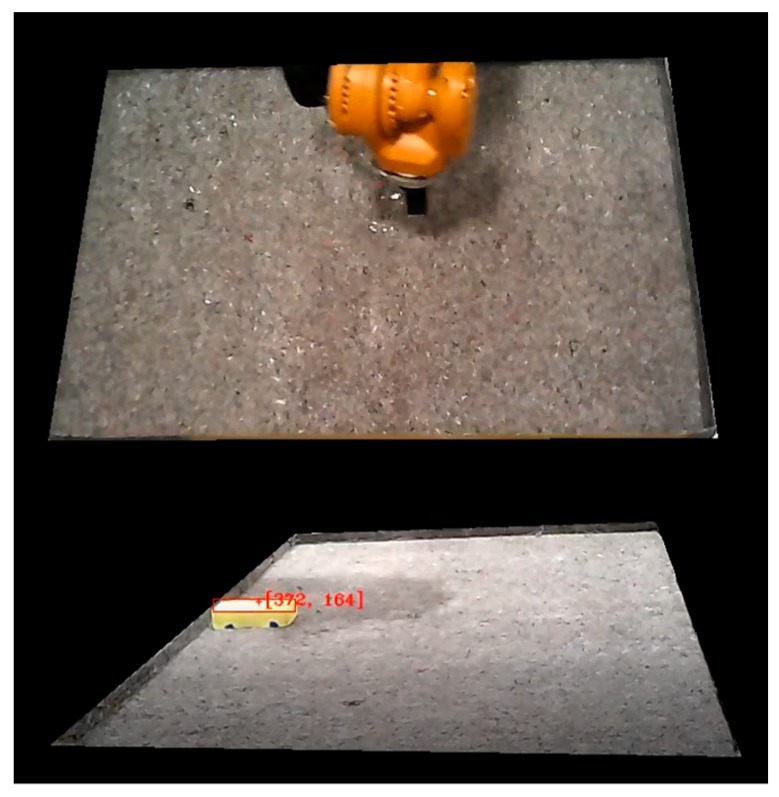
Frames from a particular moment during demonstration in which the robotic arm blocks the vision of Cam-1 (top) but Cam-2 (bottom) is able to keep tracking the obstacle.

Indeed, the machine vision was able to track the moving obstacle continuously, regardless of any challenging and dynamic conditions such as reflections, shadows and motion blur. Moreover, the issues relating to the robot intruding the field of view of Cam-1 were effectively handled by the complementary Cam-2. Figure 18 shows matching frames from Cam-1 and Cam-2 during demonstrations, showing a particular instance in which the robot intrudes the field of view in Cam-1 and completely covers the dynamic obstacle. As shown in the figure, Cam-2 was able to detect the obstacle when Cam-1 could not. Hence, the information package (Figure 6) corresponding to these frames was generated from the image of Cam-2.

### 7.3. Computing Performance

The main evaluation in this work is based on the computation time of the system and how this affects the system response. In particular, two aspects of performance are evaluated: the working cycle and system reaction time. Working cycle here refers to the time it takes for the system to update its output (refresh rate). As the different modules in the system run in parallel, the working cycle is simply determined by the module with the highest computation time. On the other hand, the reaction time can be stated as the time it takes for the system to start reacting after a given stimulus. Therefore, the reaction time requires strictly sequential execution through all stages, from initial cameras acquisition to final robot movement. In this section, the computation time per working cycle of each module and the whole integrated system is presented. Then, in the next section, an evaluation on the system reaction time is provided.

**Figure 19:**
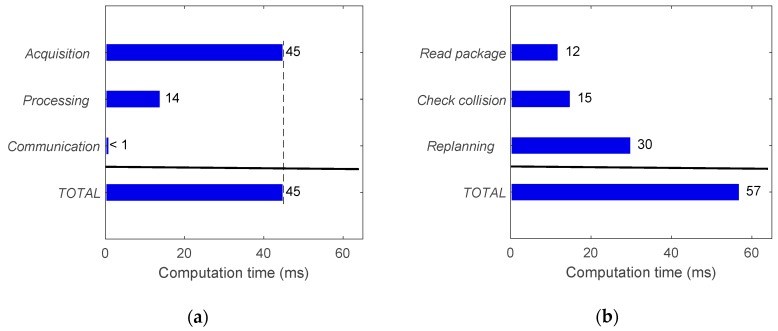
Approximated average computation times for: (**a**) Machine vision module with acquisition, image processing and communication stages running simultaneously (times do not accumulate); (**b**) Path planning module with read package, check collision and re-planning stages running in cascade, where the total time is the sum of the three stages.

Approximated average computation times for the machine vision and path planning modules are provided in Figure 19. For machine vision, the main bottleneck was frame acquisition, which took approximately 45ms to obtain a frame from the cameras. This is due to the frame rate limitations imposed by hardware. However, the image processing took only 14ms and the communication (information package preparation) was negligible. It is worth mentioning that the individual computation times in each stage of the machine vision module do not accumulate as they are implemented in parallel threads, leading to a working cycle of 45ms. On the other hand, each stage of the path planning module (Figure 19b) are executed in cascade so the total time is the sum of the individual function costs. Note that the reported computation time for path planning corresponds to the total time required from receiving sensory information to outputting a new motion plan. Finally, in relation to the robot control module, the time required for sending command positional packets was negligible as the update rate of the RSI-based approach is equal to the running frequency of the RSI context. Consequently, a new target position can be set every 4ms if necessary.

**Figure 20:**
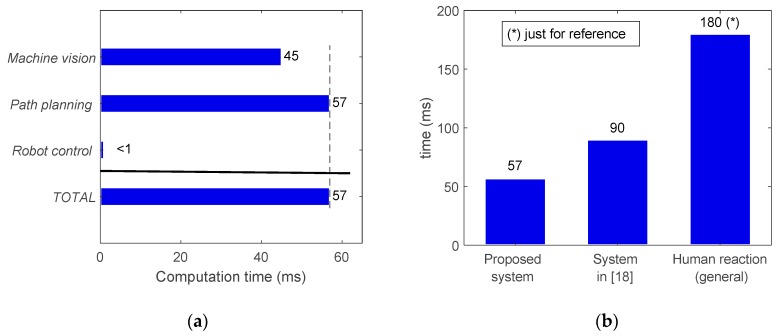
Approximated average computation times involved in: (**a**) Working cycle of the proposed system including machine vision, path planning and robot control modules running simultaneously (times do not accumulate); (**b**) Working cycle comparison between the proposed system and a different system developed in [20], including the human reaction time as a reference.

The total time required for a working cycle of the system is provided in Figure 20, including the time for the machine vision (45 ms), path planning (57 ms) and robot control (negligible) modules. As these modules run simultaneously, these times do not accumulate and the working cycle was estimated at 57 ms. The modularity of the proposed system allows a reduction in the working cycle with relation to other similar systems [20] (Figure 20b). In other words, the response of the system can be updated at a higher frequency. However, note that the working cycle frequency is not the same as the reaction time, which is evaluated in the next section.

### 7.4. Reaction Time for Human Interaction

Reaction time is the most important parameter in real-time control, since it measures the promptness of the system, and it can be defined as the latency between the stimulus and the very start of the reaction. Therefore, reaction time is crucial for robots that must possess real-time adaptive behaviors to respond to dynamic changes and/or to interact with humans. Determining the average reaction time for humans is something not straightforward, and different values are suggested in literature. Indeed, according to [44], the average reaction time for humans depends on the stimulus, being 250 ms for a visual stimulus, 170 ms for an auditory stimulus, and 150 ms for a haptic stimulus. Other references such as [20] state a general value of 180 ms for the average human reaction time. In this work, the sensing capabilities come from a machine vision implemented by means of optical cameras, so it seems reasonable to adopt as reference the time related to visual stimulus (250 ms). However, the more challenging reaction time of 180 ms [20] is also referred to as reference.

Reaction time has to be measured from the stimulus to the start of the reaction. Therefore, unlike the previously evaluated working cycle, it includes the whole computational sequence consisting of perception and related processing, motion planning and actuation such that their individual computational costs accumulate. Additionally, the reaction time for robotic systems is not only dependent on the computation times from the different stages but also on the inherent mechanical reaction time of the physical robot (intrinsic latency). While this issue is usually not addressed in literature [10,20,21], it has been taken into consideration in this work for a more complete evaluation.

Indeed, the reaction time (robot latency) of the RSI-based external control used in this work was measured 100 times through commanding the robot to move to a target from a static position (with ITRA running within MATLAB and saving robot feedback positions through the saving thread). The timestamp of the first robot feedback positional packet, reporting a deviation greater or equal to 0.01 mm from the original home position, was compared with the timestamp taken just before sending the target position to the robot. It was found that the resulting reaction time was 30 ms (±3 ms).

**Figure 21:**
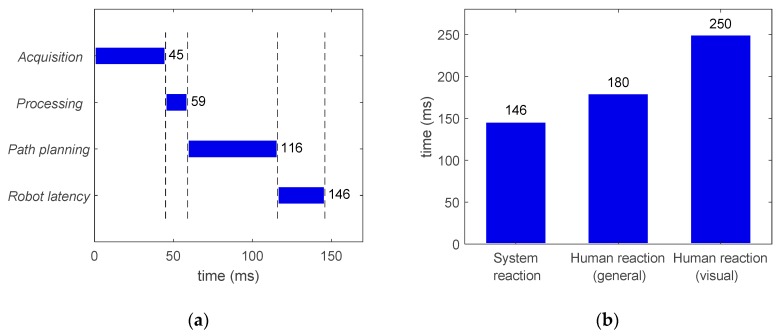
Approximated average reaction times: (**a**) The proposed system including machine vision (acquisition, processing), path planning and robot latency in sequential implementation (times do accumulate); (**b**) A comparison between the proposed system, the general human reaction time [20] and the visual human reaction time [44].

After analyzing the robot intrinsic latency, the reaction time of the proposed system was measured to be 146 ms, which consists of frames acquisition (45 ms) and image processing (14ms), path (re-)planning (57 ms), and robot latency (30 ms). This is shown in Figure 21, where the system reaction time is clearly below both the reaction time to a visual stimulus (250ms) and the reference for general human reaction time (180 ms).

## 8. Conclusions

Robotic systems are becoming more widely adopted by industries for their manufacturing processes. However, these are typically traditional systems consisting of robots following predefined tasks planned offline. A need for a strong technological shift has become apparent within industry to move towards more intelligent and autonomous systems, able to interact with their environment.

In this work, a flexible and autonomous intelligent system with environmental awareness ready for human/robot interaction is proposed and trialed on a physical demonstrator. It is based on the integration of three independent modules working in real time: (i) machine vision (smart sensing), (ii) path planning (reasoning, decision making), and (iii) robot control (movement coordination). Machine vision is based on two webcams placed off-board in fixed locations, where their frames are processed to detect obstacles by color in the HSV space. During path planning, this information is retrieved from the machine vision and used to (re-)plan a series of motion paths to enable pick-and-place operations while interacting with a dynamically-moving obstacle in the robot workspace. The planner is based on the dynamic roadmaps method, with the A* algorithm used for subsequent graph search and B-splines for path smoothing. Finally, a novel approach using RSI was developed for real-time robot control, where the target position of the robot can be updated every 4ms whilst enabling real-time trajectory modifications.

With these modules running simultaneously and communicating to each other by TCP/IP sockets, an effectively integrated system was achieved that is low-cost, highly integrated and modular. Both simulations and physical experimentation based on the KUKA KR90 R3100 robot were used to evaluate the system. From conducted trials, the system consistently executed all computations for a given working cycle in under 60ms, which is an improvement to similar cases in literature. Similarly, the system’s overall reaction time was experimentally determined to be on average 146 ms, which is indeed below the average human reaction time (180 ms). For future applications, any of the modules can be easily interchanged with other implementations for perception, planning and acting. A more dedicated image processing could be implemented for the machine vision, for example, techniques based on saliency detection and/or deep learning [45,46,47]. Additionally, obstacle detection can be extended to 3D, while other sensing capabilities can be adopted (such as laser or ultrasound) and applied to robotic tasks as required by the application whilst maintaining the modularity and advantage of the designed system.

## Figures and Tables

**Table 1:** Robot characteristics for the KUKA KR90 R3100.

Characteristics	Values
Working envelope	66 m^3^
Weight	1121 kg
Axis 1 (speed)	±185 º (105 º/s)
Axis 2 (speed)	−5 º to −140 º (101 º/s)
Axis 3 (speed)	+155 º to −120 º (107 º/s)
Axis 4 (speed)	±350 º (292 º/s)
Axis 5 (speed)	±125 º (258 º/s)
Axis 6 (speed)	±350 º (284 º/s)

**Table 2:** Three planning problems used to assess the computational efficiency of the planner.

ID	Length (mm)	Start x (mm)	Start y (mm)	Start z (mm)	End x (mm)	End y (mm)	End z (mm)
1	1643.5	10500	-6400	975	10500	-5200	975
2	1994.9	11000	-6400	975	10300	-5200	975
3	1981.9	10200	-5700	975	11200	-5700	975

**Table 3:** Computation time (ms) for path planning in simulations.

ID	Collision Checking	A* path planning	Path smoothing	Convert Cartesian	Total
1	30.3 ± 1.6	1.64 ± 0.4	3.06 ± 0.5	3.12 ± 0.2	38.1 ± 2.5
2	17.8 ± 1.3	8.00 ± 0.3	3.62 ± 0.4	3.72 ± 0.4	33.1 ± 2.3
3	38.5 ± 3.3	1.36 ± 0.6	2.42 ± 0.2	2.7 ± 0.5	45.0 ± 4.0

## Supplementary Materials

The following are available online at https://doi.org/10.15129/4df22803-2cc4-4cce-9cea-509f88f1b504, Video S1: pick place1.mp4, Video S2: pick place2.mp4.
